# Comparative Evaluation of Caudal Epidural Method to Ultrasound-Guided S1 Transforamen Block in Patient’s Lumbar Discectomy with Failed Back Syndrome Symptoms: A Double-Blind Clinical Trial

**DOI:** 10.5812/aapm-137325

**Published:** 2023-12-29

**Authors:** Masoud Hashemi, Artadokht Khoshooei, Alireza Amanollahi, Sogol Asgari

**Affiliations:** 1 Anesthesiology Research Center, Emam Hossein Hospital, Shahid Beheshti University of Medical Sciences, Tehran, Iran; 2 Clinic of Pain, Imam Hossein Hospital, Shahid Beheshti University of Medical Sciences, Tehran, Iran; 3 Trauma and Injury Research Center, Iran University of Medical Sciences, Tehran, Iran; 4 Department of Epidemiology, School of Public Health and Safety, Shahid Beheshti University of Medical Sciences, Tehran, Iran; 5 Anesthesiology Research Center, Shahid Beheshti University of Medical Scienes, Tehran, Iran

**Keywords:** Post-lumbar Surgery Syndrome, Failed Back Surgery, Transforaminal, Caudal, Radicular Pain

## Abstract

**Background:**

Post-lumbar surgery syndrome (PLSS) refers to persistent or recurrent pain following spinal surgery with an unknown cause. It is commonly associated with epidural fibrosis (EF). Some studies suggest that epidural steroid injection (ESI) can effectively alleviate pain in PLSS, particularly when targeting the S1 nerve root using S1 transforaminal epidural steroid injection (S1-TFESI). A key factor in a successful block is accurately visualizing the first dorsal sacral foramen, and the needle’s destination is the dorsal S1 foramen. Although S1-TFESI is often performed under fluoroscopy through the transforaminal route, an alternative to reduce radiation exposure is ultrasound guidance. This study aimed to compare the efficacy of ultrasound-guided caudal epidural steroid injection (CESI) and S1-TFESI in PLSS patients.

**Methods:**

A randomized double-blinded clinical trial was conducted involving 52 PLSS patients who were randomly assigned to either the CESI group or the S1-TFESI group. The patients were positioned prone. A linear transducer with a curve at a low frequency (2 - 5 MHz) was used to visualize the area. An aseptically prepared puncture site was used to insert a 2- to 5-MHz curved ultrasound probe with an ultrasound gel to identify the articular processes of the lower lumbar vertebrae and the posterior sacral surface. The probe was then positioned longitudinally to the para-sacral area, about 2 cm lateral to the midline. The articular process represented the L5/S1 level at the farthest caudal side, and the S1 posterior sacral foramen was represented by the concavity at the posterior sacral surface that was somewhat caudal. The probe was angled caudally to provide enough room for the needle to enter the S1 foramen. The injection site for the needle tip was the S1 foramen. A combination of triamcinolone (40 mg, 1 mL), normal saline (2 mL), and ropivacaine (0.2%) was administered. For TFS1 ESI, 5 mL of the combination was used. For CESI, the sacral hiatus was palpated in the prone position, and a linear high-frequency transducer was placed transversely to obtain a transverse view of the sacral hiatus. A combination of triamcinolone 40 mg and ropivacaine (0.2%) totaling 10 mL was employed. The Numerical Rating Scale (NRS) and Oswestry Disability Index (ODI) were used to assess patients’ preoperative and postoperative conditions, and adverse events were recorded. Follow-up was conducted one week and one month after the procedures.

**Results:**

In both groups, NRS and ODI scores decreased at different time points after treatment, compared to baseline (P < 0.001). The CESI group had lower median ODI scores after one week and one month, although this difference did not reach statistical significance (P = 0.334). Despite similar baseline NRS ratings, the CESI group had statistically significantly lower mean NRS scores at one week and one month (P < 0.001).

**Conclusions:**

The current study demonstrated that both CESI and TFESI treatments for PLSS following lumbar discectomy are effective and safe. These procedures can alleviate pain and reduce disability. Although the success rates of the two procedures are comparable, CESI appears to be more successful in reducing pain at the one-week and one-month follow-up.

## 1. Background

Following one or more spine surgeries, a condition known as failed back surgery syndrome (FBSS) might manifest. Failed back surgery syndrome is characterized by persistent or recurrent low back pain, with or without radicular pain (1, 2). After lumbar laminectomy with or without fusion, the incidence of FBSS is estimated to be between 10% and 40% (2, 3). Post-lumbar surgery syndrome (PLSS) is a term used to describe pain that persists or recurs following spine procedures, and it lacks a recognized cause. The development of FBSS might be influenced by various preoperative, operative, and postoperative variables (2, 3). Preoperative risk factors include litigation, worker compensation, smoking, obesity, psychological conditions, and foraminal stenosis (4, 5). Operative factors encompass inadequate decompression, excessive decompression, and surgeries performed at the wrong level. Postoperative variables include the progression of degenerative changes, altered biomechanics, muscle hypertrophy, atrophy, and spasms. Patients with FBSS typically undergo one or more surgical procedures that fail to alleviate their radicular and back pain (5, 6).

Given the high failure rate of reoperations in managing FBSS patients (7), minimally invasive interventional techniques should be considered for pain management. Studies have indicated that caudal epidural steroid injection (CESI) is an effective treatment for PLSS patients who do not respond to conventional pain-relieving medications (8, 9). Two outpatient procedures commonly used for PLSS treatment are transforaminal epidural steroid injection (TFESI) and interlaminar ESI (10).

Lumbosacral transforaminal epidural injection (TFEI) is an effective treatment for lumbosacral radiculopathy and is often performed under fluoroscopy (11, 12). However, fluoroscopy has drawbacks, including radiation exposure to the patient and the need for fluoroscopic equipment (13). Several studies have explored the efficacy of ultrasound-guided lumbosacral TFEI (13, 14). Unlike other levels of the lumbar spine, the S1 foramen is easily identifiable and accessible near the skin, making it suitable for lumbosacral TFEI treatments (15).

Lumbosacral transforaminal epidural steroid injection is a proven therapeutic method for treating lumbosacral radicular pain (16, 17). Specifically, S1 transforaminal epidural steroid injection (S1-TFESI) provides an effective nerve block for relieving pain associated with the S1 nerve root. This targeted method of epidural injection delivers a high concentration of drugs to the pathological site and spinal ganglion, yielding better results than other epidural injection techniques (18-20).

## 2. Objectives

Existing literature supports the effectiveness of ESI in managing PLSS-related pain; there is a lack of studies comparing the efficacy of different ESI techniques. Consequently, this study aimed to compare the effectiveness of CESI and TFES1 SI in treating patients with PLSS.

## 3. Methods

### 3.1. General Information

This single-center, double-blinded, prospective randomized clinical trial was conducted at the Department of Pain Medicine, Faculty of Medicine, Shahid Beheshti University of Medical Sciences, Tehran, Iran. Prior to participating in the trial, all patients were informed of its purpose and provided signed informed consent. The Ethics Committee of the Faculty of Medicine at Shahid Beheshti University of Medical Sciences approved the study (IR.SBMU.RETECH.REC.1401.875), and it was registered in the Iranian Registry of Clinical Trials (IRCT20190325043107N31).

### 3.2. Inclusion and Exclusion Criteria

The inclusion criteria were as follows: Patients between the age range of 30 and 85 years who presented with low back and lower limb radicular discomfort, who underwent previous lumbar surgery, who had specifically open, non-fusion discectomy within the last three months, who showed epidural fibrosis (EF) involving the L4, L5, or S1 nerve root on magnetic resonance imaging (MRI), and who experienced low back and leg pain that was unresponsive to conservative treatments.

The exclusion criteria were as follows: Previous surgery for more than 2-level disc herniation, on the basis of the description of a neurosurgeon in the last lumbar fusion surgery; recurrent disc herniation; sacroiliac joint pain; lumbar spondylolysis, spondylolisthesis, or scoliosis; epidural steroid injection in the past six months; bleeding tendency; systemic or local infection; pregnancy; known hypersensitivity to the administered drugs.

### 3.3. Randomization, Allocation, and Blinding Process

This study was conducted as a randomized clinical trial with a block size of four, employing a double-blinded approach (involving a statistical consultant and patients) without a control group. The study consisted of parallel groups and 2 - 3 phases, with the participation of 52 patients who underwent discectomy. The randomized clinical trial involved 52 patients who experienced lower limb radicular symptoms after discectomy surgery and were referred to Imam Hossein hospital in Tehran, Iran. These patients were randomly divided into two groups, namely the CESI group (n = 26) and the TFES1SI group (n = 26), using computer-generated random numbers for randomization.

The block random division method was used for randomization. One of the researchers created two packages, with package A representing the caudal block method and package B representing the transforaminal block method. These packages were distributed among the eligible cases using random block division (blocks of four). From the created blocks, multiple combinations were randomly selected to reach the required sample size. Numbers from 1 to 13 were assigned to the possible blocks (13 blocks) to generate a random sequence. Block numbers were then selected from a table of random numbers, determining the sequence of blocks in each group based on these numbers. Finally, random allocation was carried out with the aid of the 13 blocks of four. The initial researcher assigned the patients to study groups using blocks of four, completed the relevant checklists, and referred them to the statistical consultant. The statistical consultant remained unaware of the groups to which the patients belonged.

### 3.4. Surgical Procedure

Prior to the procedure, the patient received an 18-gauge intravenous (IV) line, and vital signs, such as heart rate, blood pressure, blood oxygen level, and electrocardiogram (ECG), were continuously monitored.

Patients were positioned in a prone manner with a cushion beneath the lower belly to reduce lumbar spine lordosis, thereby improving visibility. A linear transducer with a curved low-frequency probe (2 - 5 MHz) was utilized. Initially, the sacral cornua (SC), the apex of the sacral hiatus, and the sacrococcygeal ligament were identified using a transverse scan. Subsequently, a sterilely prepared puncture site was used to introduce a 2- to 5-MHz curved ultrasound probe with ultrasound gel to identify the articular processes of the lower lumbar vertebrae and the posterior sacral surface. The probe was then positioned longitudinally in the para-sacral area, approximately 2 cm lateral to the midline. The articular process represented the L5/S1 level at the farthest caudal side, although the S1 posterior sacral foramen was represented somewhat caudally by the concavity at the posterior sacral surface. The caudal epidural space was made visible by turning the transducer 90 degrees to examine the sacral canal in the long axis.

**Figure 1.**
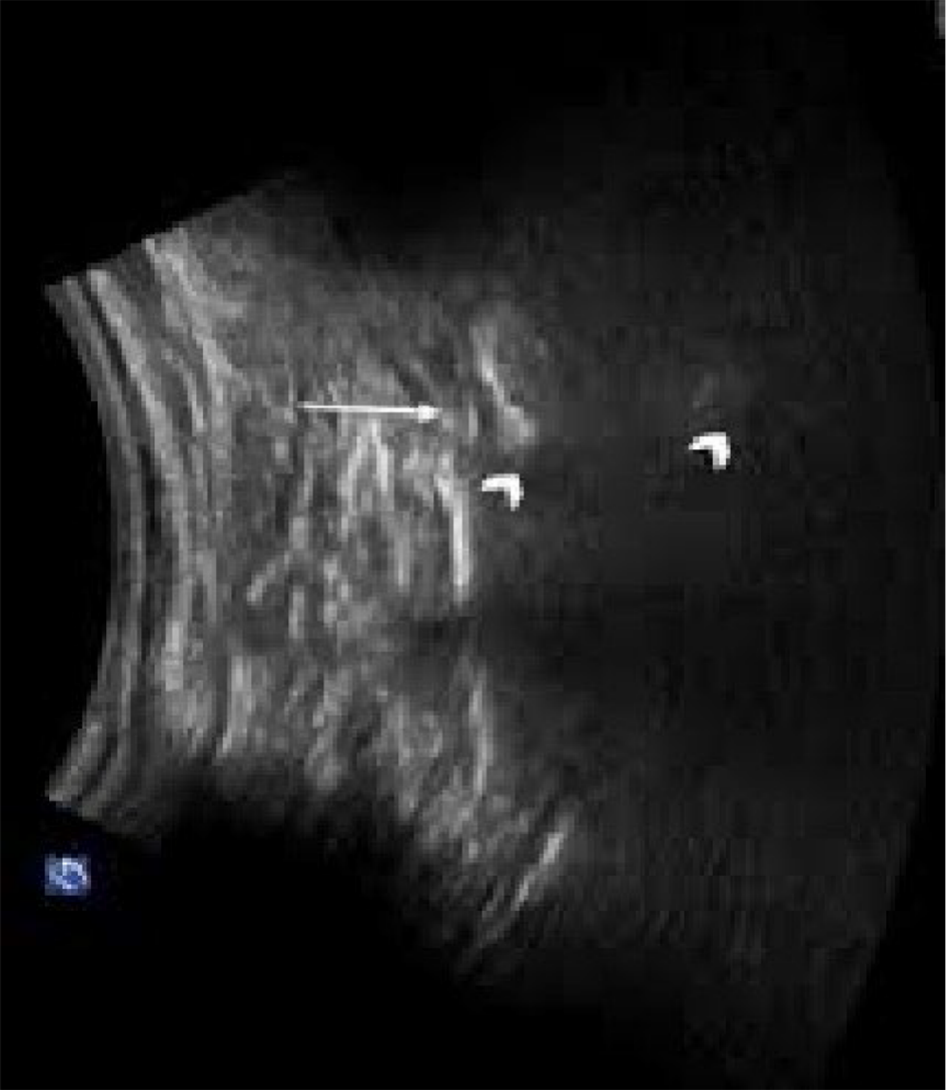
The S1 transforaminal view in ultrasound

Figure 1 shows the S1 transforaminal view in ultrasound.

**Figure 2.**
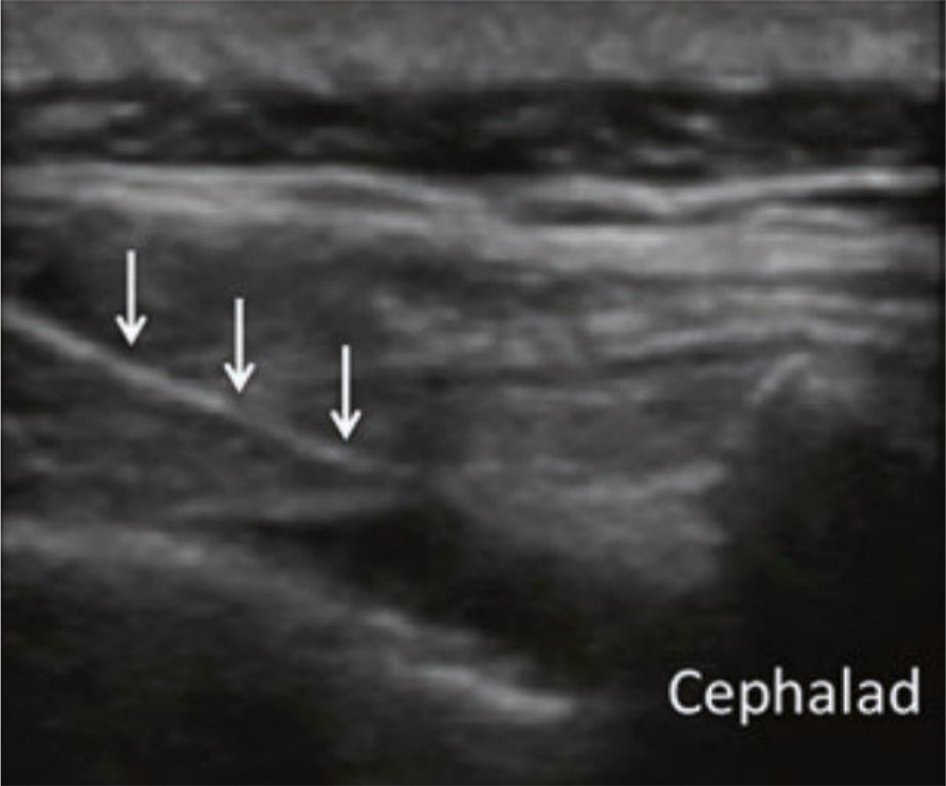
The caudal ESI ultrasound view

Figure 2 shows the caudal ESI ultrasound view.

Subsequently, the needle was inserted in a cephalad direction at a very shallow angle into the sacral canal, which appeared as a hypoechoic region between the surface of the sacrum and the thick sacrococcygeal ligament. Triamcinolone in a dose of 40 mg (1 mL), saline in 4 mL, and ropivacaine in 5 mL (0.2%) were administered. For CESI, a total of 10 mL of the combination was employed, and for TFS1 ESI, 5 mL of the combination was utilized.

### 3.5. Clinical Evaluation

All patients were followed up using the Numerical Rating Scale (NRS) and Oswestry Low Back Pain Disability Questionnaire (OSWDI) at baseline, one week, and 1 month after the procedure.

The Numerical Rating Scale is the simplest and most commonly used numerical scale in which the patient rates pain on a scale from 0 (no pain) to 10 (most severe pain).

Oswestry Low Back Pain Disability Questionnaire is a patient-completed questionnaire that provides a subjective percentage score of the level of function (disability) in activities of daily living for individuals rehabilitating from low back pain.

### 3.6. Statistical Assessment

Quantitative data were assessed for normal distribution and equality of variance. Quantitative data were presented with mean and standard deviation, although qualitative variables were reported as frequencies and percentages. The baseline characteristics of the participants in the study groups were compared using independent sample *t*-tests and chi-square tests. The mean outcomes of the study were compared using univariate analysis at each time interval, adjusting for baseline values. Finally, linear mixed model regression was employed to examine the effect of interventions during the time on the study outcomes, in addition to other covariates. The covariance matrix structure applied to the models was of the heterogeneous compound symmetry type, which showed the lowest goodness of fit (Akaike information criterion [AIC] and Bayesian information criterion [BIC]) values compared to other covariance structures. The analyses were performed using SPSS software (version 26), with significance considered at a p-value less than 0.05.

## 4. Results

**Figure 3.**
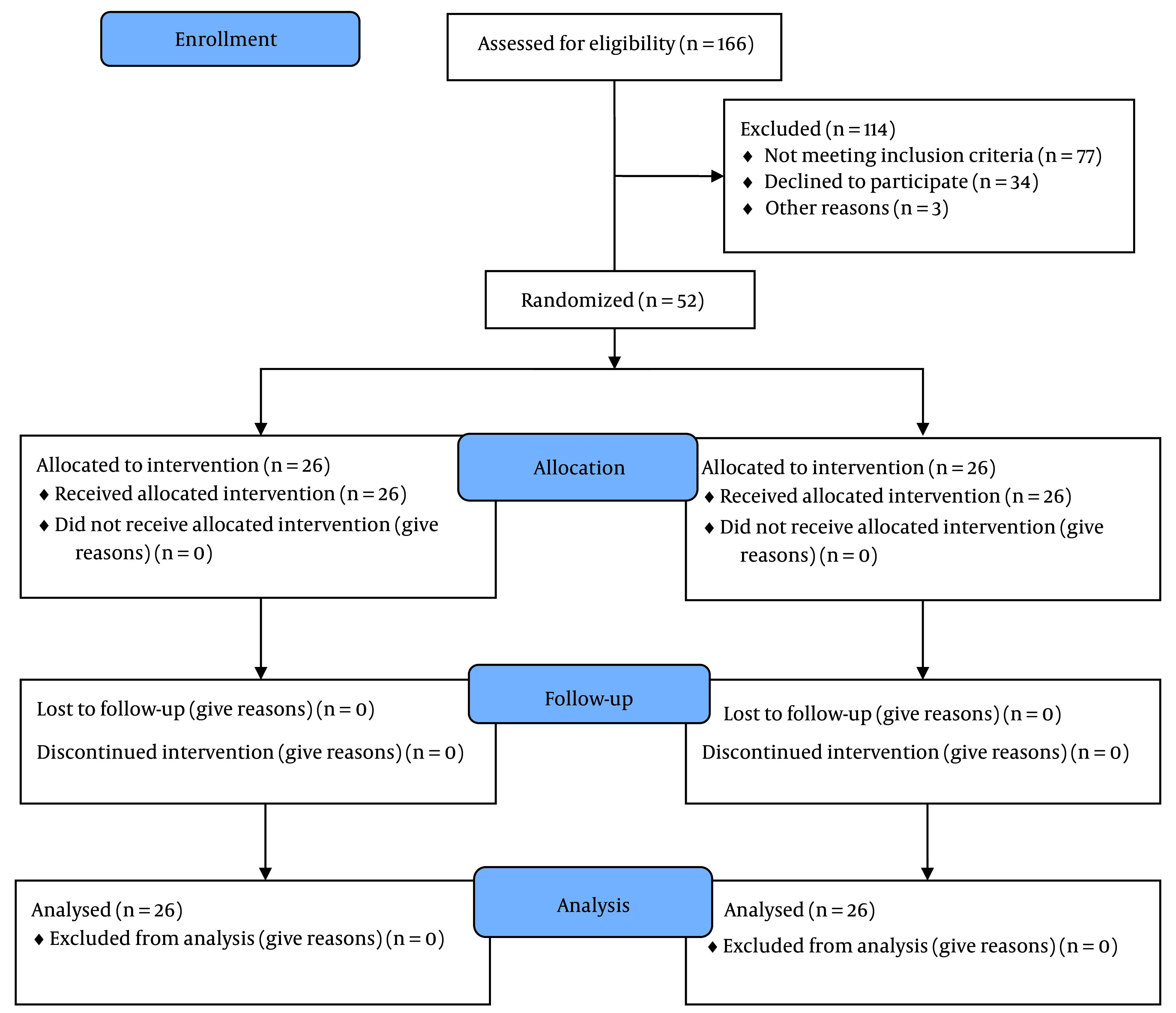
Consort flow chart of study

The analysis included 52 participants (26 from the CESI group and 26 from the S1-TFESI group), as shown in Figure 3. 

Figure 3 shows the consort flow chart of the study. In the CESI group, the mean age was 59.38 ± 13.76 years; nevertheless, in the TFESI group, the mean age was 61.27 ± 14.34 years.

Before the operation, the median NRS was 8.42 ± 0.987 and 8.65 ± 0.939 for the CESI and S1-TFESI groups, respectively. Before the surgery, the median Oswestry Disability Index (ODI) was 34.73 ± 8.13 and 32.04 ± 10.02 in the S1-TFESI and CESI groups, respectively (Table 1). 

**Table 1.** Demographic and Clinical Baseline Information ^a^

Variables	Caudal Epidural (n = 26)	S1 Transformation Block (n = 26)	P-Value
**Age (y)**	59.38 ± 13.76	61.27 ± 14.34	0.631
**Time after surgery (mon)**	10.08 ± 5.35	9.65 ± 5.09	0.775
**NRS**	8.42 ± 0.987	8.65 ± 0.939	0.391
**ODI**	32.04 ± 10.02	34.73 ± 8.13	0.293
**Gender (male)**	14 (50)	14 (50)	> 0.99
**Level (single)**	12 (48)	13 (52)	> 0.99

Abbreviations: NRS, Numerical Rating Scale; ODI, Oswestry Disability Index.

^a^ Values are expressed as mean ± SD or No. (%).

Table 1 shows the demographic details of the included cases.

**Figure 4.**
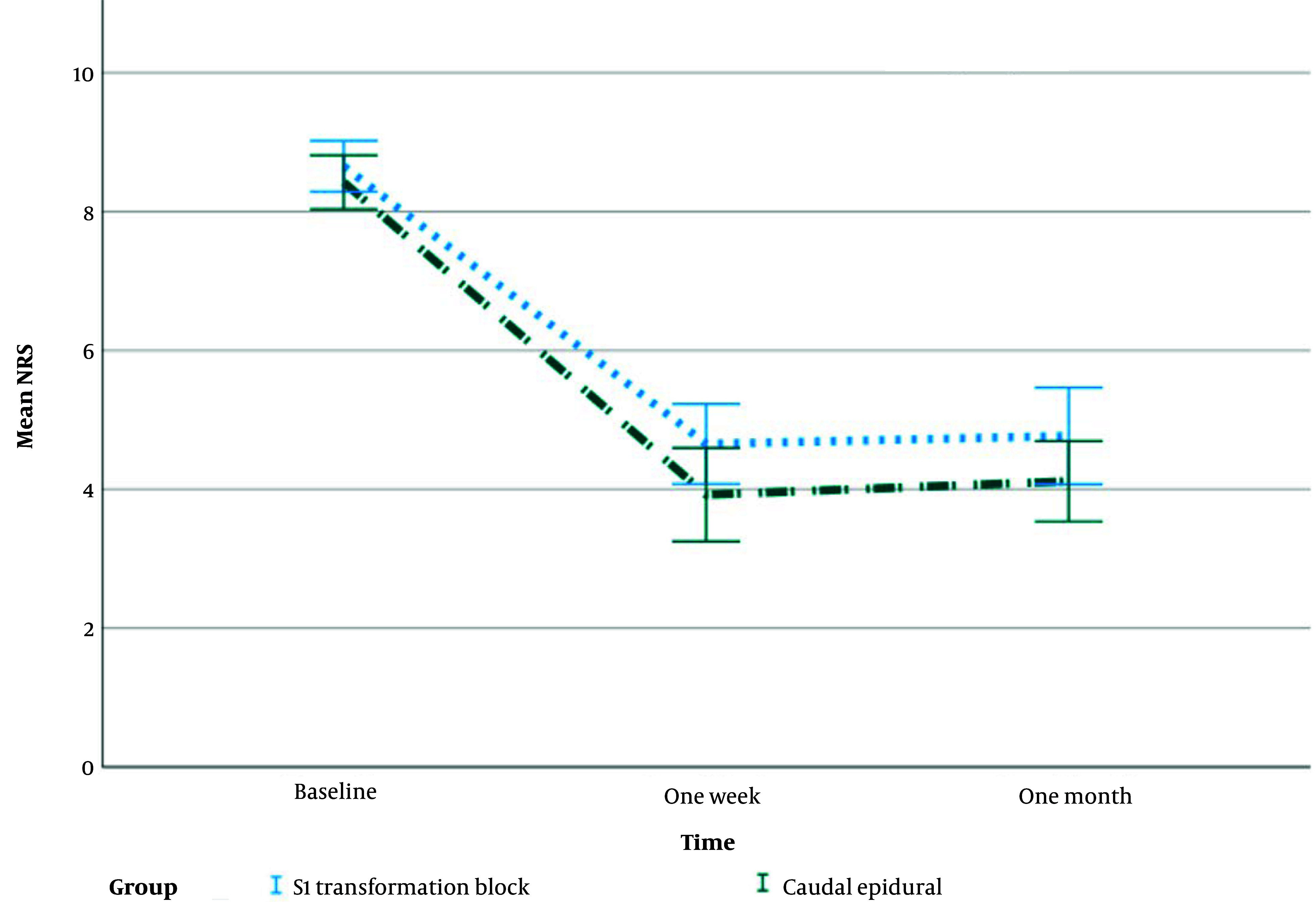
Mean changes with standard error of NRS during the study

Figure 4 illustrates the mean changes with the standard error of NRS during the study.

**Figure 5.**
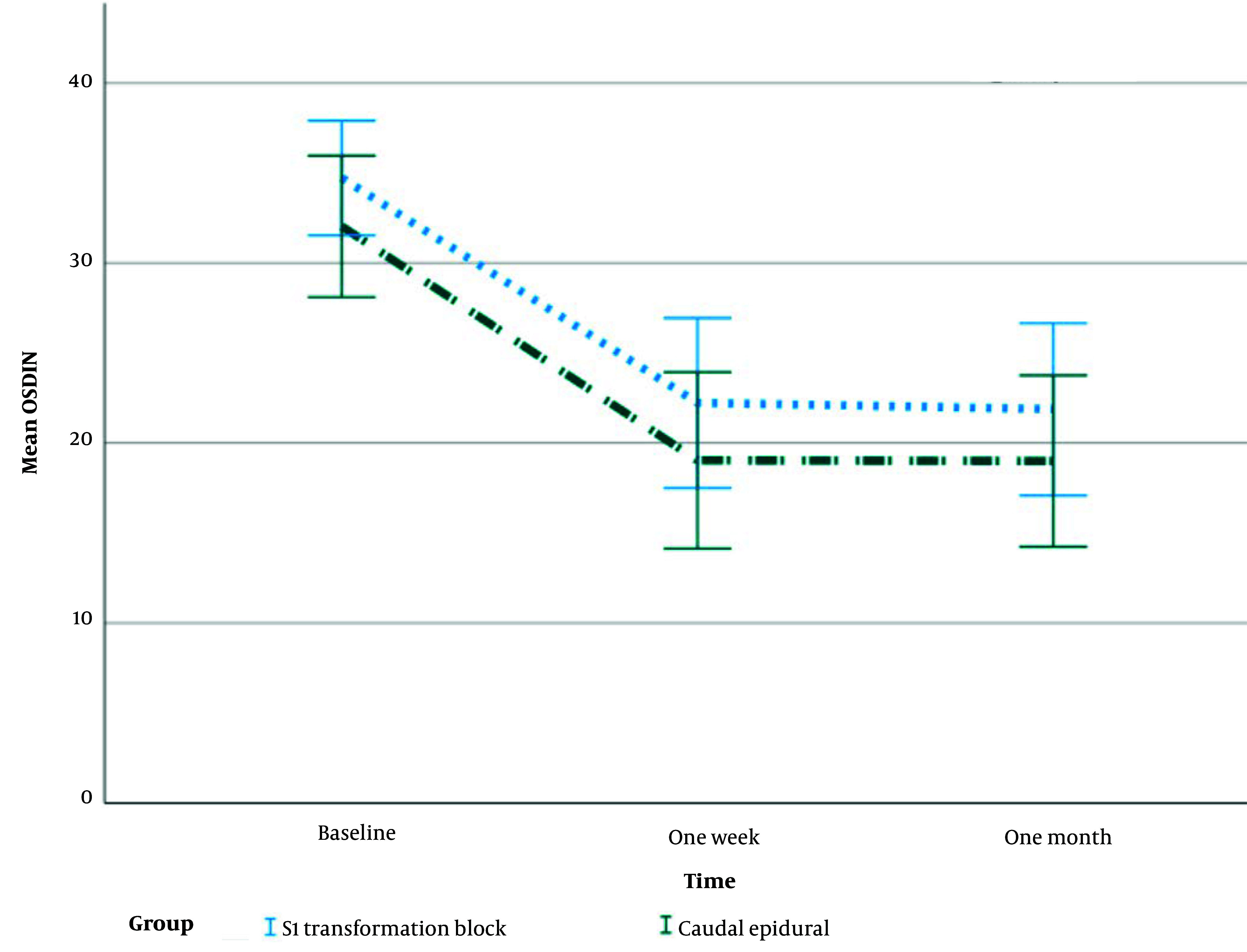
Mean changes with standard error of Oswestry Disability Index during the study

The average NRS scores in both groups declined with time, which was statistically significant (P > 0.001). Despite equal baseline NRS ratings, the CESI group had statistically significantly lower mean NRS scores at one week and one month (P < 0.001) (Figure 5, Table 2). 

**Table 2.** Effect of Covariates in Mean Changes of Numerical Rating Scale (NRS) and ODI in Follow-up

Variables	NRS		ODI	
	Coefficient (Standard Error)	P-Value	Coefficient (Standard Error)	P-Value
**Caudal epidural**	-0.12 (0.06)	0.040	-1.94 (1.99)	0.334
**S1 transformation block (ref)**	-		-	
**Follow-up**				
1 month	-0.70 (0.04)	< 0.001	-12.94 (0.81)	< 0.001
1 week	-0.73 (0.04)	< 0.001	-12.75 (0.81)	< 0.001
Baseline (ref)	-	-		
**Level**				
Two	0.02 (0.05)	0.694	3.61 (1.92)	0.063
One (ref)	-		-	
**Age**	0.007 (0.002)	0.001	0.58 (0.07)	< 0.001
**Time (after surgery)**	0.001 (0.005)	0.902	-0.15 (0.18)	0.408

Abbreviations: NRS, Numerical Rating Scale; ODI, Oswestry Disability Index.

Table 2 shows the effect of covariates in mean changes of NRS and ODI in follow-up.

Figure 5 shows mean changes with standard error of ODI during the study.

In all time points, the median ODI scores for both groups improved significantly (P < 0.001). Despite having equal baseline ODI values, the CESI group had lower median ODI scores after one week and one month, although this difference did not reach statistical significance (P = 0.334).

The median duration of symptoms in the CESI and TFESI groups was 10.08 ± 5.35 and 9.65 ± 5.09 months, respectively (Table 1), and had no significant effect on NRS and ODI after follow-up (Table 2). 

Both groups’ NRS and ODI scores increased statistically significantly with age (P < 0.001). Numerical Rating Scale and ODI scores showed an additive increase with age. The level and timing of postoperative pain onset had no statistically significant impact on NRS and ODI (Table 2). 

## 5. Discussion

In this study, individuals with PLSS who underwent discectomy for LDH were compared for the effectiveness of CESI and S1-TFESI. The findings of the present study demonstrated that both approaches had an impact on both groups’ capacity to manage pain and impairment after one week and one month following the surgery. However, after one week and one month, the CESI group showed statistically more significant improvement in NRS ratings than the S1-TFESI group. The TFESI group showed more remarkable improvement in ODI scores after 1 week than the CESI group. In the TFESI and CESI groups, the percentage of patients whose NRS-11 scores had decreased by at least 50% at one-month follow-up was 30% and 26.7%, respectively.

In the study by Akkaya et al., (21) individuals with PLSS received either ultrasound- or fluoroscopy-guided CESI. The authors concluded that CESI is a practical analgesic approach in treating PLSS, having observed significant reductions in pain and ODI scores in both groups over a 3-month follow-up period. The aforementioned findings are consistent with the results of the current study. In the present investigation, the significantly lower NRS and ODI scores of the CESI group at one week and one month might be attributed to the inability of the S1-TFESI injection to adequately deliver disability and pain relief to the epidural area where pathological changes occur.

With a low percentage of complications, CESI is the safest and simplest ESI (22). It is considered a safer method than interlaminar ESI and TFESI, making it suitable for use in patients with coagulation disorders and coagulopathy (23). In patients with PLSS, who typically experience multiple pain points, CESI offers significant advantages as it covers a wide area with a high dose in a single session (24). Mohamed et al. (25) used caudal epidural injection in their research on individuals with disc pathologies at different levels (L4-5 and L5-S1). Mohamed et al. observed no discernible difference between the groups in terms of pain relief, disability, or patient satisfaction. Based on the aforementioned findings, the current study also considered patients with distinct levels of spinal nerve root compression (L4-5 and L5-S1), in addition to disability and pain relief. Both groups showed noticeable improvements in disability and pain relief. Numerical Rating Scale scores were statistically significantly lower in the CESI group than in the S1-TFESI group at one week and 1 month.

Treatment options for FBSS, as reviewed by Amirdelfan et al., can typically be divided into five categories: Drugs, physical therapy and exercise, interventional treatments, neuromodulation, and reoperation. There is substantial evidence supporting vigorous exercise and interventional procedures, such as CESI and TFS1S, although the evidence for medications and reoperation is weak (26).

Celenlioglu et al.’s prospective study showed that both CESI and TFESI are efficient and safe methods for treating PLSS caused by EF after lumbar discectomy. The aforementioned techniques can reduce discomfort and disability. Transforaminal epidural steroid injection appears to be a more successful treatment strategy in reducing disability after a 3-week follow-up, although both approaches had similar rates of treatment success. Although similar to the present study, in terms of follow-up, NRS scores were significantly lower than ODI scores in the CESI group than in the S1-TFESI group (27).

Ultrasound-guided transforaminal epidural S1 injection with in-plane access and color Doppler imaging was the subject of a case study by Park. The patient experienced relief of referred pain, with a visual analog scale score decreasing from 5 to 1, one week after ultrasound-guided S1 TFEI, consistent with the findings of the current study (28).

In a study conducted by Akkaya et al., individuals with PLSS received either ultrasound- or fluoroscopy-guided CESI. The results showed a significant reduction in pain and ODI scores in both groups over a 3-month follow-up period, leading to the conclusion that CESI is a successful analgesic therapy for PLSS. The aforementioned findings align with the results of the present study (21-29).

Numerical Rating Scale and ODI scores were strongly correlated with age in both groups of the current study. Several factors can contribute to this correlation. As mentioned previously, the etiology of PLSS might include epidural fibrosis, perineural scarring, acquired stenosis, recurrent disc herniation, or sacroiliac and facet joint pain (30, 31).

The most common consequences of TFESI are intravascular penetration and vasovagal responses (32). In the current study, none of the participants reported any severe problems during or after the procedure.

### 5.1. Conclusions

The current study demonstrated that after lumbar discectomy, both CESI and TFESI are effective and safe treatments for PLSS. These treatments can reduce pain and disability. Caudal epidural steroid injection appears to be more successful in reducing pain at 1-week and 1-month follow-ups despite the similar treatment success rates of the two approaches. Further extensive, long-term, prospective, randomized controlled trials are needed to gain a deeper understanding of these methods.

## Data Availability

All analyzed data during this study are included in this article. Further inquiries can be directed to the corresponding author.

## Clinical Trial Registration Code:

IRCT20190325043107N31.

## References

[1] Alizadeh R, Sharifzadeh SR (2022). Pathogenesis, etiology and treatment of failed back surgery syndrome.. Neurochirurgie..

[2] Chan CW, Peng P (2011). Failed back surgery syndrome.. Pain Med..

[3] Sebaaly A, Lahoud MJ, Rizkallah M, Kreichati G, Kharrat K (2018). Etiology, evaluation, and treatment of failed back surgery syndrome.. Asian Spine J..

[4] Yeung A, Gore S (2014). Endoscopic foraminal decompression for failed back surgery syndrome under local anesthesia.. Int J Spine Surg..

[5] Knight MT, Jago I, Norris C, Midwinter L, Boynes C (2014). Transforaminal endoscopic lumbar decompression & foraminoplasty: a 10 year prospective survivability outcome study of the treatment of foraminal stenosis and failed back surgery.. Int J Spine Surg..

[6] Eichholz KM, Ryken TC (2003). Complications of revision spinal surgery.. Neurosurg Focus..

[7] Hussain A, Erdek M (2014). Interventional pain management for failed back surgery syndrome.. Pain Pract..

[8] Tao XG, Lavin RA, Yuspeh L, Bernacki EJ (2014). Implications of lumbar epidural steroid injections after lumbar surgery.. J Occup Environ Med..

[9] Manchikanti L, Sanapati MR, Soin A, Manchikanti MV, Pampati V, Singh V (2020). An updated analysis of utilization of epidural procedures in managing chronic pain in the medicare population from 2000 to 2018.. Pain Physician..

[10] Manchikanti L, Pampati V, Soin A, Sanapati MR, Kaye AD, Hirsch JA (2021). Declining utilization and inflation-adjusted expenditures for epidural procedures in chronic spinal pain in the medicare population.. Pain Physician..

[11] Koh W, Choi SS, Karm MH, Suh JH, Leem JG, Lee JD (2015). Treatment of chronic lumbosacral radicular pain using adjuvant pulsed radiofrequency: a randomized controlled study.. Pain Med..

[12] Kwak S, Jang SH, Chang MC (2021). Long-term outcomes of transforaminal epidural steroid injection in patients with lumbosacral radicular pain according to the location, type, and size of herniated lumbar disc.. Pain Pract..

[13] Sato M, Mikawa Y, Matuda A (2013). Ultrasound and electrical nerve stimulation-guided S1 nerve root block.. J Anesth..

[14] Gofeld M, Bristow SJ, Chiu SC, McQueen CK, Bollag L (2012). Ultrasound-guided lumbar transforaminal injections: feasibility and validation study.. Spine (Phila Pa 1976)..

[15] Lee BJ, Han J, Park D (2020). A video of ultrasound-guided s1 transforaminal epidural injection using color doppler: Technical reports.. Pain Pract..

[16] Manchikanti L, Buenaventura RM, Manchikanti KN, Ruan X, Gupta S, Smith HS (2012). Effectiveness of therapeutic lumbar transforaminal epidural steroid injections in managing lumbar spinal pain.. Pain Physician..

[17] Chun EH, Park HS (2015). Effect of high-volume injectate in lumbar transforaminal epidural steroid injections: A randomized, active control trial.. Pain Physician..

[18] Kim DH, Yoon DM, Yoon KB (2015). Incidence of intravascular injection and the spread of contrast media during S1 transforaminal epidural steroid injection by two approaches: anteroposterior vs oblique.. Anaesthesia..

[19] Eker HE, Cok OY, Aribogan A (2010). A treatment option for post-injection sciatic neuropathy: transsacral block with methylprednisolone.. Pain Physician..

[20] Furman MB, Butler SP, Kim RE, Mehta AR, Simon JI, Patel R (2012). Injectate volumes needed to reach specific landmarks in s1 transforaminal epidural injections.. Pain Med..

[21] Akkaya T, Ozkan D, Kertmen H, Sekerci Z (2017). Caudal epidural steroid injections in postlaminectomy patients: Comparison of ultrasonography and fluoroscopy.. Turk Neurosurg..

[22] Manchikanti L, Malla Y, Wargo BW, Cash KA, Pampati V, Fellows B (2012). A prospective evaluation of complications of 10,000 fluoroscopically directed epidural injections.. Pain Physician..

[23] Manchikanti L, Knezevic NN, Navani A, Christo PJ, Limerick G, Calodney AK (2021). Epidural interventions in the management of chronic spinal pain: American society of interventional pain physicians (ASIPP) comprehensive evidence-based guidelines.. Pain Physician..

[24] Hashemi M, Dadkhah P, Taheri M, Dehghan K, Valizadeh R (2019). Cervical epidural steroid injection: Parasagittal versus midline approach in patients with unilateral cervical radicular pain; a randomized clinical trial.. Bull Emerg Trauma..

[25] Mohamed MM, Ahmed M, Chaudary M (2007). Caudal epidural injection for L4-5 versus L5-S1 disc prolapse: is there any difference in the outcome?. J Spinal Disord Tech..

[26] Amirdelfan K, Webster L, Poree L, Sukul V, McRoberts P (2017). Treatment options for failed back surgery syndrome patients with refractory chronic pain: An evidence based approach.. Spine (Phila Pa 1976)..

[27] Celenlioglu AE, Sencan S, Bilim S, Sancar M, Gunduz OH (2022). Comparison of caudal versus transforaminal epidural steroid injection in post lumbar surgery syndrome after single-level discectomy: A prospective, randomized trial.. Pain Physician..

[28] Park D (2018). Ultrasound-guided S1 transforaminal epidural injection using the in-plane approach and color doppler imaging.. Am J Phys Med Rehabil..

[29] Kim M, Lee S, Kim HS, Park S, Shim SY, Lim DJ (2018). A comparison of percutaneous endoscopic lumbar discectomy and open lumbar microdiscectomy for lumbar disc herniation in the korean: A meta-analysis.. Biomed Res Int..

[30] Hu ZX, Han J, Sun YF, Tian XL (2022). Comparison of percutaneous endoscopic lumbar discectomy vs. minimally invasive transforaminal lumbar interbody fusion for the treatment of single-segment lumbar disc herniation: a meta-analysis.. Eur Rev Med Pharmacol Sci..

[31] Chang A, Ng AT (2020). Complications associated with lumbar transforaminal epidural steroid injections.. Curr Pain Headache Rep..

[32] Gharibo CG, Fakhry M, Diwan S, Kaye AD (2016). Conus medullaris infarction after a right L4 transforaminal epidural steroid injection using dexamethasone.. Pain Physician..

